# Confidence intervals for the COVID-19 neutralizing antibody retention rate in the Korean population

**DOI:** 10.5808/GI.2020.18.3.e31

**Published:** 2020-09-23

**Authors:** Catherine Apio, Md. Kamruzzaman, Taesung Park

**Affiliations:** 1 Interdisplinary Program in Bioinformatics, Department of Statistics, Seoul National University, Seoul 08826, Korea; 2 Department of Statistics, Seoul National University, Seoul 08826, Korea

**Keywords:** antibody, confidence interval, COVID-19, retention rate

## Abstract

The coronavirus disease 2019 (COVID-19), caused by severe acute respiratory syndrome coronavirus 2 (SARS-CoV-2), has become a global pandemic. No specific therapeutic agents or vaccines for COVID-19 are available, though several antiviral drugs, are under investigation as treatment agents for COVID-19. The use of convalescent plasma transfusion that contain neutralizing antibodies for COVID-19 has become the major focus. This requires mass screening of populations for these antibodies. While several countries started reporting population based antibody rate, its simple point estimate may be misinterpreted without proper estimation of standard error and confidence intervals. In this paper, we review the importance of antibody studies and present the 95% confidence intervals COVID-19 antibody rate for the Korean population using two recently performed antibody tests in Korea. Due to the sparsity of data, the estimation of confidence interval is a big challenge. Thus, we consider several confidence intervals using Asymptotic, Exact and Bayesian estimation methods. In this article, we found that the Wald method gives the narrowest interval among all Asymptotic methods whereas mid p-value gives the narrowest among all Exact methods and Jeffrey’s method gives the narrowest from Bayesian method. The most conservative 95% confidence interval estimation shows that as of 00:00 on September 15, 2020, at least 32,602 people were infected but not confirmed in Korea.

## Introduction

Several unexplained pneumonia cases have been successively discovered in Wuhan, Hubei province of China, in early December 2019, which have been confirmed to be severe acute respiratory syndrome caused by a novel coronavirus 2 (severe acute respiratory syndrome coronavirus 2, SARS-CoV-2) and has spread rapidly across the globe [1]. The spread of coronavirus disease 2019 (COVID-19) became a global threat and the World Health Organization (WHO) declared COVID-19 a global pandemic on March 11, 2020 [2]. As of September 18, 2020, there were a total of 30,358,087 confirmed cases and 950,635 deaths from COVID-19 worldwide [3].

No specific therapeutic agents or vaccines for COVID-19 are available yet [4]. Accurate and fast diagnosis of the SARS-CoV-2 is one of the most important methods used to isolate patients with COVID-19 and stop the spread of the epidemic, as well as save people’s lives [5] The current recommendations for laboratory diagnosis of COVID-19 from the Center for Disease Control (CDC) are that clinicians coordinate this testing with local public health authorities and/or the CDC. Viral nucleic acid detection using real-time reverse-transcription polymerase chain reaction assay, like that developed for the diagnosis of SARS-CoV, was developed and used for rapid detection of SARS-CoV-2 and remains the gold standard for diagnosis of COVID-19 [6].

Although several antiviral drugs, such as the nucleotide analogue remdesivir and favipiravir, are under investigation as treatment agents for COVID-19, the antiviral efficacy of these drugs is not yet known [4,7]. In addition to vaccine development and approaches that directly target the virus or block viral entry, treatments that address the immunopathology of the infection have become a major focus. The use of convalescent plasma was recommended as an empirical treatment during outbreaks of Ebola virus in 2014 and the treatment of Middle East respiratory syndrome coronavirus in 2015 [8,9]. This approach was effective with other viral infections such as SARS-CoV, H5N1 avian influenza, and H1N1 influenza [10], through a single convalescent plasma transfusion [11-13]. Treatment of severe infections of patients of influenza A(H1N1) 2009 pandemic with convalescent plasma was associated with reduced respiratory tract viral load, serum cytokine response, and mortality [10]. Also, the study involving 80 patients with SARS showed that administration of convalescent plasma was associated with a higher rate of hospital for patients who received the convalescent plasma than with patients who did not receive the plasma [10,14].

In addition, high-throughput platforms, such as the large-scale single-cell RNA sequencing of B cells (enriched for B cells that produce antibodies directed at the SARS-CoV-2 spike glycoprotein) from patients who are convalescent, have allowed the identification of SARS-CoV-2-specific neutralizing antibodies [6]. Neutralizing antibodies is said to play an important role in virus clearance and have been considered as a key immune product for protection or treatment against viral diseases. Convalescent plasma containing identified SARS-CoV-2–specific neutralizing antibodies has already been used to treat a small number of patients with severe disease, and preliminary results show clinical improvement in 5 of 5 critically ill patients with COVID-19 who had developed acute respiratory distress syndrome [6,10]. All these findings raise the hypothesis that use of convalescent plasma transfusion is beneficial in patients infected with SARS-CoV-2 and solidifies the importance of large-scale antibody testing for COVID-19 [15].

A COVID-19 antibody test, also known as a serology test, is a blood test that can detect if a person has antibodies to SARS-CoV-2. An antibody test checks for antibodies (proteins made by the immune system to fight infections like viruses and may help to ward off future occurrences by those same infections) in the blood. Human bodies make antibodies when we catch an infection to help fight the infection [16,17]. If coronavirus antibody is present in the blood, it's likely he/she had the virus before. The value of antibody tests is currently limited to (1) answering the question of whether someone has had the virus before, (2) providing data and a greater understanding on the spread of the virus [18]. Also, given the unknown scale of asymptomatic infections (infected patients without symptoms), there is a pressing need for serological diagnosis to represent the real number of COVID-19 infected patients which determines the true extent of infection in a given country [19]. Serological tests are known to be in use in Europe, United States, Japan and other developed countries to figure out how many people are infected with the potentially deadly virus [20]. For example, results from Spain’s final stage of a nationwide antibody study shows that Spain’s antibody retention rate is believed to be 5% [21] while London, Stockholm, and Tokyo have a retention rate of 17%, 7.3%, and 0.1%, respectively [22]. While viral RNA-based testing for acute infection is the current standard, surveying antibody protection is a necessary for discovering the real extend of coronavirus infections in a population and for return to social normality [19,20].

Other importance for COVID-19 antibody testing include; to identify donors with high-neutralizing titers for convalescent plasma for therapy and define correlates of protection from SARS-CoV-2 [19].

## Methods

### Materials

Recently, Korea Centers for Disease Control and Prevention (KCDC) announced the discovery of neutralizing antibodies for COVID-19 from two investigative screening surveys carried in South Korea. The first antibody screening results reported; 0 neutralizing antibodies were discovered out of 1,555 serum samples collected for antibody titer from subjects who participated in the Korea National Health and Nutrition Examination Survey (KNHANES) from April 21 to June 16 from 14 cities and provinces in South Korea excluding Daegu (which was the city for the major COVID-19 outbreak in Korea in early March), Daejeon and Sejong (0/1,555) and neutralizing antibodies was identified in only one sample out of 1,500 serum samples collected from May 25 to 28 from patients visiting a medical institution in southwestern Seoul (1/1,500) [22]. The second antibody results reported that neutralizing antibodies were confirmed in only one sample out of 1,400 serum samples collected for antibody titer from subjects who participated in KNHANES from June 10 to July 13 in 13 cities and provinces including Daegu, Daejeon, and Sejong (1/1,400).

The above reported dataset only captures a sample proportion but does not provide its confidence interval. This kind of result reporting can mislead the public especially to people without any statistical knowledge. Based on these sample datasets, we will use inferential statistics for deciding for whole population in Korea about COVID-19 antibody screening studies. Because our sample data is sparse, point estimation (i.e., sample proportion) gives some misleading interpretations, for example; 0/1,555 sample proportion shows that there are no neutralizing antibodies present in the Korean population, and so it is good to report point estimation along with proper interval estimation. In paper, we present intervals (95% confidence intervals) for the above reported Korean COVID-19 neutralizing screening antibody results using known Asymptotic, Exact, and Bayesian estimation methods.

### Methods

As one method doesn’t give the optimal confidence interval range, we present confidence intervals calculated using different methods that can apply the point estimate results above. There are many methods available for calculation of confidence intervals for various parameters and those methods are mainly divided into three different type of estimation techniques, such as asymptotic estimation, exact estimation, and Bayesian estimation. In this section, we have reviewed the likelihood function for binomial proportion as well as the methods for confidence interval.

#### Likelihood function for binomial parameter

Let we conduct *n* iid Bernoulli experiments with probability of success *π* and find *y* successes. Then the likelihood function can be defined as

$$L(\pi )=\left(\overset{n}{y}\right)\pi^{y}{\left(1-\pi \right)}^{n-y},\;\mathrm{for}\;y=0,\cdots ,\;n$$

and the log-likelihood function is

$$l(\pi )=\;\ln(\overset{n}{y})+\mathrm{yln\pi}+(n-y)\ln(1-\pi )$$

Maximum likelihood estimator of *π* is $\hat{\pi }=y/n$, the sample proportion of success for n trials, and the standard error of $\hat{\pi }$ is $\hat{\pi }(1-\hat{\pi })/n$.

#### Asymptotic estimation (large sample approximation)

Confidence intervals can be obtained by inverting the association test [23]. For instance, a 95% confidence interval for the population proportion *π* is the set of *π*_0_ for which test of *H*_0_:*π*=*π*_0_ has p-value exceeding 0.05 [23]. Wald, Score, and Likelihood-ratio are the three main asymptotic methods for estimating confidence intervals as described below.

Wald confidence interval

The 100(1-α)% wald confidence interval is

$$\left\{\pi :\frac{\left|\hat{\pi }-\pi \right|}{\mathrm{SE}}<z_{\alpha /2}\right\}\Rightarrow (\hat{\pi }-z_{\frac{\alpha }{2}}(SE),\;\hat{\pi }+z_{\frac{\alpha }{2}}(SE))$$

where *z*_*α*/2_ is the z-score form the standard normal distribution with right tailed probability *α*/2.

Likelihood-ratio-based confidence interval (LR)

The 100(1-*α*)% likelihood-ratio-based confidence interval is

$$\left\{\pi :\left|2\left[L\left(\hat{\pi }\right)-L(\pi )\right]\right|<\chi_{1}^{2}(\alpha )\right\},$$

where *L*(*π*) is the maximized value of likelihood function under *H*_0_:π = *π*_0_ and *L*($\hat{\pi }$) is the likelihood function calculate at the ML estimate $\hat{\pi }$, and $x_{1}^{2}(\alpha )$ is the 100(1-*α*) percentile of the chi-square distribution with 1 degree of freedom.

Score-based confidence interval

The 100(1-α)% score-based confidence interval is

$$\left\{\mathrm{\pi :}\frac{\left|u(\pi )\right|}{I{\left(\pi_{0}\right)}^{\frac{1}{2}}}<z_{\alpha /2}\right\},$$

where *u*(*π*) is the score function derived from the log-likelihood function.

#### Exact estimation

For both small and large to moderate samples, population proportion inference can occur both near 0 or 1, and both are not good. In such cases, asymptotic methods may have inadequate performance and provide quite different confidence intervals [23]. Therefore, we use alternative estimation techniques, such as exact sample inference and Bayesian sample inference. For exact estimation, we use the Clopper-Pearson and the mid p-value methods. Clopper-Pearson interval is based on inverting the tailed binomial tests for forming confidence intervals [24]. For a binomial data with parameter *π* (success), the endpoints are the solution of

$$\sum_{k=y}^{n}(\overset{n}{k})\pi^{k}{\left(1-\pi \right)}^{n-k}=\frac{\alpha }{2},\sum_{k=0}^{y}(\overset{n}{k})\pi^{k}{\left(1-\pi \right)}^{n-k}=\frac{\alpha }{2},$$

and the lower bound is 0 when *y*=0 and the upper bound is 1 when *y*=*n*. Unfortunately, with the discrete probability distributions, it is usually not possible for a p-value to achieve the desired significance level exactly [24], so we use the mid p-value

Mid p-value:

For small samples of discrete data, it seems sensible to use adjustment of exact methods based on the mid p-value [25]. For a test statistic *T* with observed value *t*_0_ and one-sided alternative hypothesis, the mid p-value is obtained by

$$\text{Mid}\;p-\text{value}=\frac{1}{2}p(T=t_{0})+p(T>t_{0})$$

where probability, *p* calculated from the null distribution. The two-sided mid p-value is

$$\text{Mid}\;p-\text{value}=2[\frac{1}{2}p(T=\left|t_{0}\right|)+p(T>\left|t_{0}\right|)].$$

#### Bayesian estimation

Aforementioned (asymptotic estimation and exact estimation) approaches are known as the frequentist approach and requires a random process that produces the observed data [26]. That is, the parameter value is assumed to be unknown, but a fixed quantity and obtained from the observed data. Recent years have been seen with increasing popularity of this Bayesian approach, which considers the parameter is a random quantity and whose value can be described by a probability distribution, known as *prior distribution* and fixed data. Bayesian approach combines the prior with observed data to create a posterior for the parameters using the Bayes equation;

p(πy)=p(yπ)p(π)p(y)

where *π* is the unknown parameter, *y* is the observed data and the denominator *p*(*y*) is the marginal probability function of the data, which is a constant with respect to *π*. The Bayes equation then simplifies to

p(πy)∞p(yπ)p(π)

The above equation gives the posterior probability of *π*, *p*(*π*│*y*), as a function of likelihood p(*y*│*π*) and prior *p*(*π*). Therefore, we need to choose the prior information, which is the most difficult aspect in Bayesian approach. If there is lack of prior information one can use uniform prior, which can be got from literature if a pilot study has been conducted, which turns out to be a non-informative prior. The most popular non-informative choice of prior is Jeffrey’s prior, defined as

-E(∂2lnf(yπ)∂π2)1/2

In binomial setting, the Jeffrey’s prior for binomial data is *π*~Beta ($\frac{1}{2},\frac{1}{2}$). Alternatively, when there is no prior information Beta(1,1) prior known as uniform prior (U(0,1)) can be considered.

Because the parameter *π* is a random variable in Bayesian techniques, it allows for making ideal statements concerning the probability of the parameter and confidence intervals. This confidence interval contains most of the posterior distribution and is known as the posterior interval or credible interval.

## Results

Table 1 presents the 95% confidence intervals for the first antibody results using only the KNHANES samples and for the total population in South Korea. The first two columns show the methods and the next two columns the 95% confidence interval for antibody retention rate in the samples. The final two columns represent the estimated 95% confidence interval of antibody carriers in Korean population by multiplying the total number of Korean population (51,780,579 people) with the antibody ratio (the proportion of samples with neutralizing antibodies provided as confidence intervals) from September 19, 2020. Note that this estimation was derived from a simple random sampling assumption, while the antibody sample does not represent the total Korean population.

From Table 1, the Wald method fails to provide confidence interval. LR gives the minimum upper bound which is 63,690 and it also provides narrower confidence intervals among all types of confidence intervals’ methods. Score, Exact and Uniform methods gives similar interval.

Table 2 presents the 95% confidence intervals for the first antibody results using only the Seoul samples and for the total population in South Korea. Table 3 presents the 95% confidence intervals for the first antibody results using both Seoul and KNHANES samples (1,555 + 1,500) and the total population in South Korea. Table 4 presents the 95% confidence intervals for the second antibody results using only the KNHANES samples (1,440) and for the total population in South Korea. Table 5 presents the 95% confidence intervals for the sum of the first and second antibody results using the KNHANES + Seoul + KNHANES samples and for the total population in South Korea. The first two columns show the methods and the next two columns the 95% confidence interval antibody ratio in the total samples (1,555 + 1,500 + 1,440).

For all cases, the Wald method gives the narrowest interval and smallest upper bound values, except in Table 1 (102,008, 50,227, 104,079, and 54,887, respectively) among all the asymptotic estimation methods. mid p-value method among exact estimation and Jeffrey’s method among Bayesian estimation, all give the smallest interval. Jeffrey’s and mid p-value methods have similar intervals while Score and Uniform methods have similar interval results. Bayesian Jeffrey’s prior gives better interval than Uniform Prior.

From Tables 1–5, confidence intervals are quite different depending on the method used. We think this inconsistency might be due to sparsity of data and small sample sizes. It is well known that sparsity causes the parameter estimates to fall near the boundary of the parameter space (for example, in proportion parameter value near to 0 or 1). As a result, the asymptotic methods such as the Wald method suffered from the convergence problem. In this sparse situation, either exact methods or Bayesian methods provide more reasonable confidence intervals. With modern computational power, it is not difficult to use exact inference for confidence interval directly from the binomial distribution without using large sample approximation to normality [23]. Due to discreteness, two exact methods provide different result. In Bayesian methods, Jeffrey’s prior provided better results than the uniform prior because it uses prior information for the scales of measurement, while the uniform prior does not use any prior information at all. Also, note that when the sample size was largest (Table 5), all methods provided more similar confidence intervals. In other words, as the sample size increases, all methods are expected to provide quite consistent confidence intervals.

In summary, as the sample size increases, the confidence interval become narrower. That is, as the sample size increases, more accurate estimation of antibody ratio is possible. In the confidence interval, the lower bound can be replaced by the number of confirmed patients through an actual test. Among the upper bound, the smallest value provides a conservative interpretation while the largest value does a more aggressive interpretation. Subtracting today's cumulative number of confirmed cases from the smallest upper limit can be interpreted as the minimum number of cases that were infected but not confirmed. As of 00:00 on September 15, 2020, at least 32,602 (= 54,887–22,285) people were infected but not confirmed. This should be interpreted as having a high probability of cumulative infection.

## Discussion

Statistical inference is a kind of process that helps us in making decisions about unknown population based on information contained in a sample taken from the population. There are two types of statistical inference: point estimation and hypothesis testing. The most important aspect of statistical inference is estimation of the unknown parameter, which is a procedure for finding the value of the unknown parameter by using the sample observations. For example, the sample means are used to estimate the population mean, sample proportion are used to estimate the population proportions, etc. An estimate of population parameter can be expressed in two ways: point estimate and interval estimate. A point estimate is a single number that is used to estimate an unknown population parameter. It is not much useful unless some information regarding possible error of estimate is associated with the estimate. For example, the sample proportion $\hat{\pi }$ and we expect that this sample proportion is a good estimate of the population proportion. But the sample proportion vary from sample to sample and thus sampling error may be associated with the estimate. For a given sample proportion, the amount of sampling error is not known, so the standard error can be used as an estimate for the average amount of error in sample proportion. The total proportion cannot be identified with certainty based on only sample proportion without standard error. Therefore, instead of a point estimate, an interval or a range of values which is likely to contain the population parameter, which is known as *interval estimation*, must be provided. The major advantage of using interval estimation is that it provides a range of values with known probability of capturing the population parameter. Because we recognize sampling error, the point estimate has low confidence while interval estimates overcomes this problem by using interval estimation techniques which is based on point estimate and margin of error. Thus, it is important to provide a point estimate along with its standard error or confidence intervals. Due to having more advantage of interval estimation relative to simple point estimation. In this article, we report different type of confidence interval for the sparse COVID-19 antibody test results from the Korean population.

Unfortunately, our study has several limitations. The current sample size for Korean studies is too small for accurate estimation of antibody test of Korean population. For estimation of antibody carriers in Korean population, we simply assumed that our antibody samples were derived from a simple random sampling assumption, because any detailed information of the samples is not available. Furthermore, the antibody samples may not represent the total Korean population well. To make more accurate estimation of antibody carriers in the Korean population, a much large sample size is required to represent the Korean population well. Then, with a further detailed demographic information of subjects and sampling information, more accurate sampling design-based inference can estimate the total number of antibody carriers in Korean population along with its 95% confidence intervals. In addition, there is no detailed information available for antibody test kits. Thus, in our estimations, it is expected that the kits should have high specificity, to accurately diagnose the subject without the COVID-19 antibody as negative, rather than high sensitivity to diagnose the subjects with the antibody as positive. No sensitivity and specificity information was considered in our analysis.

## Figures and Tables

**Table 1.** 95% Confidence intervals (CIs) for the first antibody results using the KNHANES samples only and for the total population in South Korea

Method	Sample 95% CI (antibody retention rate)		Total population 95% CI (antibody carriers)	
	Lower	Upper	Lower	Upper
Asymptotic estimation				
Wald^a^	0.00000	0.00000	0.00	0.0000
Score	0.00000	0.00246	0.00	127,380
Likelihood ratio	0.00011	0.00123	5,696	63,690
Exact estimation				
Exact	0.00000	0.00236	0.00	122,202
MidP	0.00000	0.00193	0.00	99,937
Bayesian estimation				
Uniform	0.00000	0.00237	0.00	122,720
Jeffrey’s	0.00000	0.00161	0.00	83,367

a Fails to provide CI.

**Table 2.** 95% Confidence intervals (CIs) for the first antibody results using only samples from Seoul area and for the total population in South Korea

Method	Sample 95% CI (antibody retention rate)		Total population 95% CI (antibody carriers)	
	Lower	Upper	Lower	Upper
Asymptotic estimation				
Wald	0.00000	0.00197	0.00	102,008
Score	0.00012	0.00377	6,214	195,213
Likelihood ratio	0.00005	0.00292	2,589	151,199
Exact estimation				
Exact	0.00009	0.00472	4,660	244,404
MidP	0.00010	0.00310	5,178	160,520
Bayesian estimation				
Uniform	0.00016	0.00371	8,285	192,106
Jeffrey’s	0.00000	0.00311	0.00	161,038

**Table 3.** 95% Confidence intervals (CIs) for the first antibody results using both KNHANES + Seoul sample size only and for the total population in South Korea

Method	Sample 95% CI (antibody retention rate)		Total population 95% CI (antibody carriers)	
	Lower	Upper	Lower	Upper
Asymptotic estimation				
Wald	0.00000	0.00097	0.00	50,227
Score	0.00000	0.00185	0.00	95,794
Likelihood ratio	0.00000	0.00144	0.00	74,564
Exact estimation				
Exact	0.00000	0.00232	0.00	120,131
MidP	0.00010	0.00160	5,178	82,849
Bayesian estimation				
Uniform	0.00000	0.00182	0.00	94,241
Jeffrey’s	0.00000	0.00153	0.00	79,224

KNHANES, Korea National Health and Nutrition Examination Survey.

**Table 4.** 95% Confidence intervals (CIs) for the second antibody results using only KNHANES sample portion and for the total population in South Korea

Method	Sample 95% CI (antibody retention rate)		Total population 95% CI (antibody carriers)	
	Lower	Upper	Lower	Upper
Asymptotic estimation				
Wald	0.00000	0.00201	0.00	104,079
Score	0.00001	0.00392	518	202,979
Likelihood ratio	0.00000	0.00308	0.00	159,484
Exact estimation				
Exact	0.00000	0.00491	0.00	254,243
MidP	0.00010	0.00310	5178	160,520
Bayesian estimation				
Uniform	0.00016	0.00386	8285	199,873
Jeffrey’s	0.00000	0.00324	0	167,769

KNHANES, Korea National Health and Nutrition Examination Survey.

**Table 5.** 95% Confidence interval (CI) using the sum of both the first and second antibody results sample size and for the total population

Method	Sample 95% CI (antibody retention rate)		Total population 95% CI (antibody carriers)	
	Lower	Upper	Lower	Upper
Asymptotic estimation				
Wald	0.00000	0.00106	0.00	54,887
Score	0.00001	0.00162	518	83,885
Likelihood ratio	0.00000	0.00137	0.00	70,939
Exact estimation				
Exact	0.00001	0.00178	518	92,169
MidP	0.00010	0.00110	5,178	56,959
Bayesian estimation				
Uniform	0.00014	0.00161	7,249	83,367
Jeffrey’s	0.00000	0.00143	0.000	74,046

## References

[1] Li Q, Guan X, Wu P, Wang X, Zhou L, Tong Y (2020). Early transmission dynamics in Wuhan, China, of novel coronavirus-infected pneumonia. N Engl J Med.

[2] WHO director-general’s opening remarks. Geneva: World Health Organization, 2020. Accessed 2020 Sep 19. Available from: https://www.who.int/dg/speeches/detail/who-director-general-s-opening-remarks-at-the-media-briefing-on-covid-19---11-march-2020

[3] Daily number of new reported cases of COVID-19 by country worldwide. Solna: European Centre for Disease Prevention and Control, 2020. Accessed 2020 Sep 19. Available from: https://opendata.ecdc.europe.eu/covid19/casedistribution/csv

[4] Wu Z, McGoogan JM (2020). Characteristics of and important lessons from the coronavirus disease 2019 (COVID-19) outbreak in China: summary of a report of 72314 cases from the Chinese Center for Disease Control and Prevention. JAMA.

[5] Xiang F, Wang X, He X, Peng Z, Yang B, Zhang J (2020). Antibody detection and dynamic characteristics in patients with COVID-19. Clin Infect Dis.

[6] Cao X (2020). COVID-19: immunopathology and its implications for therapy. Nat Rev Immunol.

[7] Lu H (2020). Drug treatment options for the 2019-new coronavirus (2019-nCoV). Biosci Trends.

[8] Tang YW, Schmitz JE, Persing DH, Stratton CW (2020). Laboratory diagnosis of COVID-19: current issues and challenges. J Clin Microbiol.

[9] Chen L, Xiong J, Bao L, Shi Y (2020). Convalescent plasma as a potential therapy for COVID-19. Lancet Infect Dis.

[10] Shen C, Wang Z, Zhao F, Yang Y, Li J, Yuan J (2020). Treatment of 5 critically ill patients with COVID-19 with convalescent plasma. JAMA.

[11] Zhou B, Zhong N, Guan Y (2007). Treatment with convalescent plasma for influenza A (H5N1) infection. N Engl J Med.

[12] Hung IF, To KK, Lee CK, Lee KL, Chan K, Yan WW (2011). Convalescent plasma treatment reduced mortality in patients with severe pandemic influenza A (H1N1) 2009 virus infection. Clin Infect Dis.

[13] Burnouf T, Radosevich M (2003). Treatment of severe acute respiratory syndrome with convalescent plasma. Hong Kong Med J.

[14] Cheng Y, Wong R, Soo YO, Wong WS, Lee CK, Ng MH (2005). Use of convalescent plasma therapy in SARS patients in Hong Kong. Eur J Clin Microbiol Infect Dis.

[15] Long QX, Liu BZ, Deng HJ, Wu GC, Deng K, Chen YK (2020). Antibody responses to SARS-CoV-2 in patients with COVID-19. Nat Med.

[16] Antibody test to check if you’ve had coronavirus. London: NHS, 2020. Accessed 2020 Sep 19. Available from: https://www.nhs.uk/conditions/coronavirus-covid-19/testing-and-tracing/antibody-test-to-check-if-youve-had-coronavirus/

[17] Antibody (serology) testing for COVID-19: information for patients and consumers. Silver Spring: Food and Drug Administration, 2020. Accessed 2020 Sep 19. Available from: https://www.fda.gov/medical-devices/coronavirus-covid-19-and-medical-devices/antibody-serology-testing-covid-19-information-patients-and-consumers

[18] Coronavirus (COVID-19): antibody testing. London: Department of Health and Social Care, 2020. Accessed 2020 Sep 19. Available from: https://www.gov.uk/government/publications/coronavirus-covid-19-antibody-tests/coronavirus-covid-19-antibody-tests

[19] Muruato AE, Fontes-Garfias CR, Ren P, Garcia-Blanco MA, Menachery VD, Xie X (2020). A high-throughput neutralizing antibody assay for COVID-19 diagnosis and vaccine evaluation. Nat Commun.

[20] S. Korea begins coronavirus antibody tests. Seoul: Yonhap News Agency, 2020. Accessed 2020 Sep 19. Available from: https://en.yna.co.kr/view/AEN20200630007300320

[21] Spanish antibody study shows 5% of population exposed to coronavirus. New York: Reuters, 2020. Accessed 2020 Sep 19. Available from: https://www.reuters.com/article/us-health-coronavirus-spain-study-idUSKBN2471AL

[22] 1 vs. 3,055: Koreans’ rate of Covid-19 antibodies. Seoul: Korea Biomedical Review, 2020. Accessed 2020 Sep 19. Available from: https://www.koreabiomed.com/news/articleView.html?idxno=8732

[23] Agresti A (2013). Categorical Data Analysis.

[24] Clopper CJ, Pearson ES (1934). The use of confidence or fiducial limits illustrated in the case of the binomial. Biometrika.

[25] Agresti A (2018). An Introduction to Categorical Data Analysis.

[26] Dobson AJ, Barnett AG (2018). An Introduction to Generalized Linear Models.

